# Can OpenCap deliver valid and reliable kinematic data for motion analysis? A systematic review and three-level meta-analysis

**DOI:** 10.5114/biolsport.2026.154942

**Published:** 2025-11-03

**Authors:** Salih Çabuk, Süleyman Ulupınar, İzzet İnce, Serhat Özbay

**Affiliations:** 1 Erzurum Technical University, Sport Sciences Faculty, Department of Coaching Education, Erzurum, Türkiye; 2 Ankara Yıldırım Beyazıt University, Sport Sciences Faculty, Department of Coaching Education, Ankara, Türkiye; 3 Erzurum Technical University, Sport Sciences Faculty, Department of Physical Education and Sports, Erzurum, Türkiye

**Keywords:** OpenCap, Validity, Reliability, Three-level meta-analysis, Markerless motion capture systems

## Abstract

Markerless motion capture systems have gained increasing interest as practical alternatives to gold-standard references systems in clinical and sports contexts. This systematic review and three-level metaanalysis aimed to evaluate the criterion validity of OpenCap and to systematically summarize the available evidence regarding its reliability. A literature search was conducted across Web of Science, PubMed, Scopus, and EBSCO. Among the 12 studies included in the systematic review, 11 provided sufficient data for the metaanalytic synthesis of criterion validity, encompassing 184 participants, from which 640 effect sizes (ES), 230 Fisher’s Z values, and 1087 root mean square error (RMSE) values were obtained. OpenCap demonstrated a statistically significant, yet practically trivial effect compared to criterion devices (ES = -0.140; p = 0.021). Fisher’s Z transformation indicated a good-to-excellent correlation with criterion devices (r = 0.845; p = 0.005). The pooled RMSE was 5.877°, which decreased to 5.197° after sensitivity analysis and further to 4.940° following trim-and-fill adjustment. In terms of reliability, test-retest consistency generally ranged from moderate to very good across many joint angles and tasks, although marked variability was observed in certain task-joint combinations, particularly in high-velocity movements and complex joint actions. OpenCap, as a smartphonebased markerless motion capture system, can provide valid and acceptable kinematic measurements when compared to criterion devices. However, its performance varies depending on task complexity and joint-specific demands, underscoring the need for evaluation across diverse populations, a wider range of task types, and within standardized methodological frameworks.

## INTRODUCTION

Motion capture systems are widely used in clinical and sports contexts for various purposes, such as analysing movement techniques, improving skill training (e.g., through real-time feedback for movement retraining), and monitoring rehabilitation or training processes [1, 2]. Additionally, motion analysis is often a key component of screening tests to identify athletes at risk of lower extremity injuries, enabling the development of personalized training programmes [3–7]. Threedimensional (3D) marker-based motion capture systems are considered the gold standard in sports science for kinematic measurements of the whole body and joint levels. However, their effectiveness is influenced by extensive setup requirements, such as marker placement on participants, the need for specialized clothing, camera calibration, and the expertise training required to conduct data collection processes [8]. In addition, high costs and the need for complex laboratory setups further complicate their applicability; limitations in portability also restrict their areas of use [2, 9–12]. These challenges in using marker-based systems encourage many clinicians working in sports medicine and biomechanics to explore alternative methods with high ecological validity to obtain the same kinematic data [13, 14]. Furthermore, the time factor is one of the most significant limitations of marker-based systems, prompting researchers to seek alternative methods for athlete and patient assessments [1]. In contrast, artificial intelligence (AI) supported markerless motion capture systems stand out with their ability to estimate 3D poses from two-dimensional (2D) red-green-blue (RGB) video cameras, offering the potential to overcome many limitations of traditional laboratory-based assessments [15]. These 2D pose estimation algorithms can detect anatomical landmarks or joint centre positions in a single video and derive an individual’s posture in each video frame or image [16]. Over the past decade, various 2D pose estimation algorithms, such as OpenPose, Theia3D, DeepLabCut, DeepPose, and DeeperCut, have been published [17–21]. The ability to quickly collect data from numerous individuals makes markerless motion capture systems a promising alternative for large-scale real-world applications. The increasing widespread use and development of these systems demonstrate that a detailed understanding of their measurement properties is of critical importance for effective use. These properties, such as the capacity of systems to obtain measurement results that are close to true values and to acceptably and validly assess the structures they aim to measure (agreement/ accuracy), as well as their ability to generate consistent measurements (reliability) [1, 2, 22], are of critical importance for effective and dependable use in clinical settings. The knowledge gained about the system’s measurement properties can enable clinicians to make informed decisions when selecting equipment for their own practices. In addition, the ability of computer vision and artificial intelligence models to precisely track different body positions and speeds may vary depending on the tasks [23]. This situation stands out as a factor that can significantly affect the validity and reliability levels of markerless motion capture systems [24]. The fact that the validity and reliability levels of these systems in analysing dynamic tasks commonly used in sports and clinical settings have not yet been fully clarified creates uncertainty regarding the widespread adoption and effective use of this technology.

OpenCap (Stanford, USA) is an open-source, web-based, and markerless motion capture system [25]. Using two iOS devices and a laptop, this system processes kinematic data through cloud-based software and outputs the result. Compared to traditional marker-based laboratory systems, OpenCap is low-cost, easy to set up, and requires minimal maintenance, making it convenient for use in non-laboratory environments. By eliminating the need to place markers on the body, it enables the analysis of natural movements in sport-specific settings, making the system a promising tool for both clinical and sports applications [26]. In a pioneering study in this field, Uhlrich et al. introduced the OpenCap system and took the first steps of validation by comparing kinematic data obtained using two iPhones with the marker-based system, which is the reference standard in the industry. When the two systems were compared during various activities such as walking, squatting, standing up from a chair, and drop jumps (DJs) in ten healthy individuals, the RMSE range across lower-extremity joint angles was found to be 2.0–10.2° [25].

The accessibility offered by OpenCap through its low-cost, portable, and markerless structure holds significant potential in various fields such as clinical applications, sports science, and academic research. This system is noteworthy for enabling movement analysis in natural environments by largely eliminating the time, cost, and setup challenges of traditional marker-based systems [27–30]. However, alongside these advantages, OpenCap also presents several practical limitations that may constrain its wider adoption. The system currently requires relatively high/new generation quality iOS devices (iPhone/iPad), which may limit accessibility in some settings. Its reliance on cloud-based processing makes stable and high-speed internet a prerequisite, potentially reducing usability in field environments with limited connectivity. Additionally, the transmission of movement data to cloud servers raises concerns regarding data privacy and security (encryption and Stanford compliance measures have been implemented), particularly in clinical applications subject to strict regulatory frameworks. Finally, the available data export formats may restrict seamless integration with other biomechanical analysis software, necessitating additional conversion steps [23, 25]. These practical challenges highlight the importance of critically evaluating OpenCap not only in terms of its potential benefits but also its current limitations when considering broader implementation. The reliable use of this technology in practical applications is directly related to the validity and reliability of its kinematic measurement outputs. Although recent literature includes studies investigating the validity of the OpenCap system across different types of movements and usage scenarios, the findings are not yet sufficiently clear or generalizable due to methodological diversity and limited sample sizes. This increases uncertainty for health professionals, sports scientists, and researchers when evaluating the suitability of the system. Valid and reliable movement analysis has a critical importance in clinical decision-making processes. It ensures the individualized and precise planning of rehabilitation protocols and the informed making of return-to-sport decisions, thereby optimizing sports performance outcomes and increasing the effectiveness of rehabilitation interventions [27–30]. Therefore, a study that examines the criterion validity levels of kinematic measurements obtained with the OpenCap system and systematically summarizes the available evidence on its reliability would fill an important gap in the field. In this context, the aim of this research is to evaluate the criterion validity of the OpenCap markerless motion capture system and to present its reliability characteristics systematically.

## MATERIALS AND METHODS

The protocol was registered on the Open Science Framework (OSF) platform, and all files regarding the study process were shared (https://osf.io/qwmsp; Registration DOİ: https://doi.org/10.17605/OSF.IO/KDT38). This systematic review was performed in accordance with the guidelines outlined in the Preferred Reporting Items for Systematic Reviews and Meta-Analyses (PRISMA).

### Literature search strategy

In this study, four electronic databases (Web of Science, PubMed, Scopus, and EBSCO) were used for the literature search. Google Scholar was also used in the follow-up search to identify additional studies that were not contained in the above databases. The literature search was initiated on November 24, 2024, and concluded on March 7, 2025. The search term used was “OpenCap” AND (“Validity” OR “Reliability” OR “Accuracy” OR “Agreement”). Additionally, the reference lists of the included studies were examined for other relevant studies. Two independent authors (S.Ç. and S.U.) screened the titles and abstracts, and articles with the potential for inclusion in the study were read in full for further evaluation.

### Inclusion and exclusion criteria

The study selection was conducted based on the Participants, Intervention, Comparators, Outcomes, and Study Design (PICOS) approach [31]. Articles published in different languages did not meet the eligibility criteria; only articles written in English were included in this study. The details of the inclusion and exclusion criteria for the study are provided in Table 1.

**TABLE 1:** Inclusion and exclusion criteria.

	Inclusion Criteria	Exclusion Criteria
**Population**	Studies involving human participants using OpenCap for motion analysis across various movement tasks (e.g., CMJ, SJ, running, gait analysis).	Studies that do not include human participants or use OpenCap for purposes unrelated to motion analysis.

**Intervention**	Studies assessing the validity and reliability of OpenCap compared to criterion devices across different movement tasks and various joint angles.	Studies that do not include a direct comparison between OpenCap and a recognized gold-standard motion capture system, or studies that only report OpenCap’s measurements without validation against a reference device.

**Comparator**	Studies that include a comparator group using criterion devices (three-dimensional motion capture system, force plate, and optoelectronic system).	Studies without an appropriate comparator group (studies without a benchmark reference system).

**Outcome**	Studies reporting Pearson correlation (r), ICC values, RMSE, or the mean and standard deviation values of both the criterion device(s) and OpenCap in different movement modalities and joint angles.	Studies that do not include statistical measures related to the criterion validity or reliability of OpenCap.

**Study Design**	Studies exclusively assessing validity and reliability of OpenCap	Randomized controlled trials (RCTs), interventionbased studies, longitudinal studies, and crosssectional studies.

**Additional Criteria**	Full-text original pre-print articles	Review articles, case studies, conference abstracts, conference papers, and M.Sc. or Ph.D. theses.

### Data extraction

The extracted data included the (a) authors, (b) year of publication, (c) sample size, (d) sample characteristics (age, body mass, and height), (e) study design, (f) OpenCap and criterion devices and their specifications (sampling frequency in Hz, number of cameras, and specifications of commercial smartphones used etc.), (g) activity pattern [jump-based or motion-based, limbs (left or right), and various joint angles and movement units, etc.], (h) mean and standard deviation for both OpenCap and the criterion device, (i) validity outcomes [Pearson correlation coefficient (r/ρ), intraclass correlation coefficient (ICC), means and standard deviations for RMSE, and mean absolute error (MAE)], and (j) reliability outcomes [r, ICC, standard error of measurement (SEM), and minimal detectable change (MDC)]. Two authors (S.Ç. and S.U.) independently extracted data from the selected articles using a pre-defined form created in Microsoft Excel (Microsoft Corporation, Redmond, WA, USA). In cases where discrepancies arose regarding the extracted data, they were resolved through consensus in consultation with a third author (İ.İ.).

### Methodological quality and risk of bias

The methodological quality of each included study was assessed using a modified Downs and Black assessment [32]. This assessment was based on five key domains: (1) reporting, (2) external validity, (3) internal validity-bias, (4) internal validity-confounding, and (5) statistical power. Items were evaluated using a binary scoring system of one (1) or zero (0): A score of one (1) indicated that the criteria were met, whereas a score of zero (0) denoted that the criteria were not met or could not be determined [33, 34]. In accordance with recommendations in the literature, specific threshold values were established for the assessment of study quality. Accordingly, studies scoring ≥ 50% were classified as “fair quality”, those scoring ≥ 70% as “good quality”, and those scoring ≥ 90% as “excellent quality”. Conversely, studies with scores below 50% were categorized as “poor quality” [35]. A total of 14 domains were identified to evaluate the quality of reporting for studies included in this review:

1. Was the hypothesis or study aim clearly described?
2. Were the main outcomes to be measured clearly described in the Introduction or Methods section?
3. Were the participant characteristics (e.g., age, sex, anthropometrics) clearly detailed?
4. Was the intervention procedure thoroughly described?
5. Were the main findings of the study clearly described?
6. Did the study provide estimates of the random variability in the data for the main outcomes?
7. Were the subjects asked to participate in the study representative of the entire population from which they were recruited?
8. Were the statistical tests used to assess the main outcomes appropriate?
9. Was compliance with the measurement protocol consistent and reliable across all participants?
10. Were the main outcome measures used accurate (valid and reliable)?
11. Were any of the results a result of p-hacking/data-dredging?
12. Was there adequate adjustment for confounding in the analyses from which the main findings were drawn?
13. Were losses of patients to follow-up taken into account?
14. Did the study have sufficient power to show reliability and/or validity? Was there a power calculation?

Two authors (S.Ç. and S.U.) independently assessed the methodological quality of the studies included in this systematic review and three-level meta-analysis. In the case of a disagreement between the two authors (S.Ç. and S.U.), the third author (İ.İ.) made the final decision. If the third author was unable to reach a definitive conclusion, the fourth author (S.Ö.) conducted the final evaluation and determined the ultimate decision. Additionally, inter-rater agreement between the two assessors was calculated using Cohen’s kappa coefficient, with a 95% confidence interval (95% CI) [36]. Cohen’s kappa coefficient was categorized as follows: values below 0.40 indicated poor agreement, those ranging from 0.40 to 0.75 were considered fair to good agreement, and values above 0.75 represented excellent agreement [37]. Beyond evaluating methodological quality, the researchers also conducted a risk of bias assessment (Figure 2). The ROBINS-I assessment tool was used to evaluate the risk of bias in non-randomized studies. The criteria established by the researchers were adopted as the evaluation criteria [38].

### Statistical analysis

The criterion validity of OpenCap in comparison to criterion devices were assessed across multiple movement modalities [e.g., countermovement jump (CMJ), squat jump (SJ), running, and gait analysis] and various joint angle regions (e.g., hip, knee, and ankle). The traditional meta-analysis approach assumes that the observed effect sizes (ESs) should be independent of each other [39]; however, this is not the case in this study. Therefore, when non-independent ESs are present, meaning that nested ESs exist within a study, a threelevel meta-analysis method was applied to account for this dependency [40]. The three-level meta-analysis models the variance arising from sample sizes (Level 1), the variance of different ESs within the same study (Level 2), and the variance of ESs across studies (Level 3) [41]. The three-level meta-analysis was conducted using a randomeffects model with restricted maximum likelihood (REML) estimation to minimize the Type I error rate. The criterion validity of the OpenCap was assessed through two complementary approaches: (1) determination of agreement/accuracy with criterion devices via RMSE and (2) evaluating correlation with criterion devices using Fisher’s Ztransformed correlation coefficients. In addition to these, Hedges’ g ESs were calculated. The negative or positive nature of ES values indicates the direction and significance of measurement differences between OpenCap and criterion devices. Specifically, a negative or positive ES reflects the direction of systematic bias (underestimation or overestimation) in OpenCap’s measurements compared to criterion devices, as well as the magnitude of this bias. ES was interpreted according to the following reference ranges: trivial (< 0.20), small (0.20–0.59), moderate (0.60–1.19), large (1.20–1.99), or very large (≥ 2.00) [42–45]. Given the need to classify reported r values, a single study could contribute to multiple independent data pools depending on the reported statistical outcomes and measured parameters for validity. The weighting of individual point estimates was based on sample size. In this context, point estimates were variance-stabilized using Fisher’s Z-transformation [46].

$$\text{Fisher’s }{\text{Z}}_{\text{r}}=0.5\times \ln\frac{1+\text{r}}{1-\text{r}}$$ (1)

$${\text{v}}_{\text{z}}=\frac{1}{\text{n}-3}$$ (2)

$${\text{SE}}_{\text{r}}=\sqrt{{\text{v}}_{\text{z}}}$$ (3)

$$\text{Summary r}=\frac{{\text{e}}^{2\text{Z}}-1}{{\text{e}}^{2\text{Z}}+1}$$ (4)

Here, represents the sample size, denotes the standard error, and indicates the summary Fisher’s Z value [47]. The data were backtransformed into r values for reporting purposes. The r values were interpreted as follows: no relationship (< 0.250), weak relationship (0.250–0.500), moderate to good relationship (0.500–0.750), or good to excellent relationship (≥ 0.750) [48].

RMSE represents the magnitude of systematic and random errors between systems while providing a straightforward and interpretable measure of prediction accuracy [14, 16, 18, 23, 49]. In this context, RMSE has been analysed as one of the primary metrics for evaluating the measurement validity between OpenCap and the criterion devices [50, 51]. RMSE values are expressed in degrees (°).

The I^2^ percentages (i.e., the proportion of total variance distributed across each level) were used to assess the heterogeneity of the pooled ES. The I^2^ statistic was interpreted based on the following reference values: low heterogeneity (< 25%), moderate heterogeneity (26–75%), and high heterogeneity (> 75%) [52]. The risk of publication bias was assessed by examining the symmetry of the funnel plot, and potential asymmetries were confirmed using the extended Egger’s test [53]. Egger’s test is based on a regression analysis in which standardized ESs are regressed against a measure representing precision, such as the standard error of the correlation coefficients. A statistically significant regression coefficient in Egger’s test indicates a relationship between ESs and sampling variance, which suggests the presence of publication bias. When evidence of publication bias was detected, Duval and Tweedie’s “trim and fill” procedure was applied to determine whether adjustments to the estimates were necessary due to the presence of missing studies [54].

The meta-analysis results were interpreted using the fit metrics. The significance level was set at p < 0.05. Subgroups were meticulously established to thoroughly investigate the nuances of various factors influencing the outcomes. These subgroups were categorized based on two key parameters: jumping-based and motion-based tasks. Statistical results not included in the meta-analytic synthesis were systematically reported to support interpretation. To assess the robustness of the results, sensitivity analyses were conducted by removing one study at a time [46, 55]. Statistical analyses were conducted using the “metafor” package in R (v 4.2.1; R Core Team, https://www.r-project.org/). Forest plot graphs were generated using GraphPad Prism version 10 (GraphPad Software, San Diego, CA, USA).

## RESULTS

### Characteristics of the included studies

**FIG. 1:**
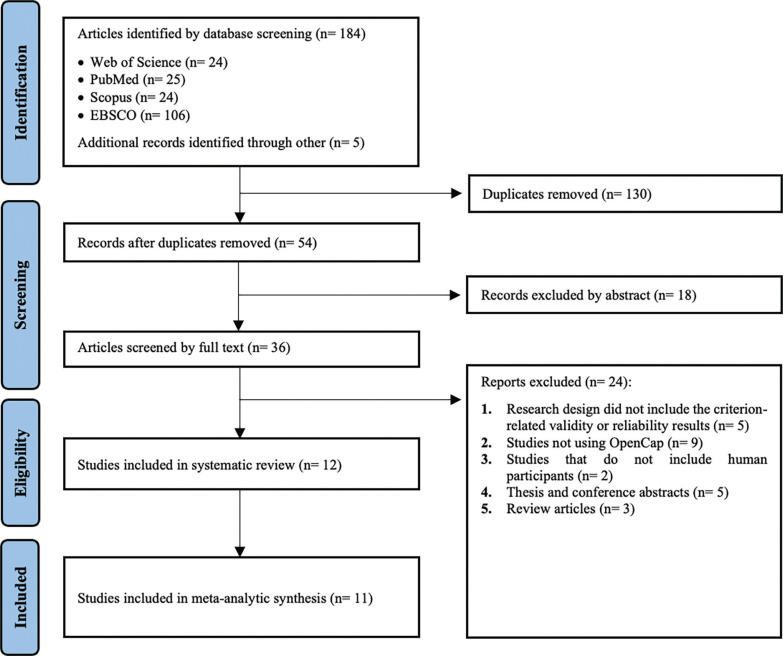
PRISMA flow diagram.

The database search process identified a total of 184 articles. After removing duplicates (n = 130), 54 articles remained for eligibility assessment. Subsequently, the reference lists and citations of the eligible studies were reviewed to identify additional relevant studies, resulting in the identification of 5 further articles. Two authors (S.Ç. and S.U.) reviewed the article titles and abstracts according to the criteria presented in Table 1. Of the initial 54 articles, 18 were excluded as irrelevant to the study scope following the screening of their abstracts. The full texts of the remaining 36 articles were then assessed, and 24 articles were excluded for various reasons (e.g., the research design did not include validity or reliability results, or the studies did not involve human participants). Consequently, 12 studies met the inclusion criteria for the systematic review, and 11 were incorporated into the meta-analytic synthesis assessing the criterion validity of OpenCap. Additionally, two studies were systematically assessed for reliability outcomes. Of these systematically evaluated studies, one was Lima et al. [2], which was included in the meta-analytic synthesis for criterion validity, but its reliability outcomes were interpreted systematically. The other study was Horsak et al., which was solely interpreted systematically [56]. Details of all these studies are provided in Supplementary Material (link). An overview of the screening process, illustrated using a PRISMA flow diagram, is presented in Figure 1.

**FIG. 2:**
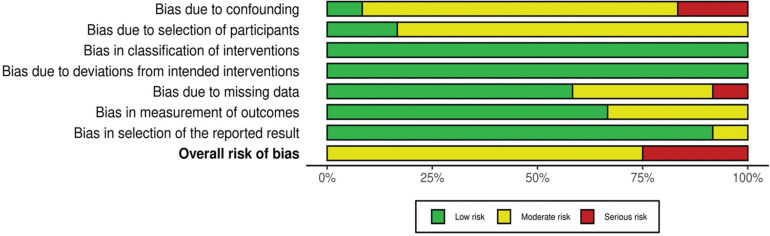
Risk of bias in studies.

Considering the 12 included studies, a total of 203 individuals were enrolled in this review (age range: 18 to 35 years; 108 males and 89 females; gender was not specified in the study by Peng et al.) [57]. The most commonly used criterion devices were the three-dimensional motion capture system [11, 27, 58, 59] and the force plate [26]. In four studies, both the three-dimensional motion capture system and the force plate were employed concurrently [2, 16, 23, 25, 57]. Only one study used an optoelectrical system [60]. Furthermore, the studies incorporated a variety of tasks, categorized into jump-based tasks (e.g., CMJ, SJ, DJ, bilateral (BDJ) and unilateral DJ (UDJ), forward hop, lateral hop, triple vertical hop, side step, side hop) [2, 26, 27] and motion-based tasks (e.g., gait, cycling, walking, running, timed up and go, sit-tostand) [11, 16, 23, 56–58]. Some studies combined both jumpbased and motion-based tasks [25, 59, 60]. Regarding the number of cameras used for the three-dimensional motion capture system, this study reports a range of 8 to 17 cameras. The frequencies of these systems vary between 100 and 250 Hz. The frequencies of force plates vary between 1000 and 2000 Hz (Tables 4 and 5).

### Methodological quality and risk of bias

No articles were rated as “poor quality”. Of the total 12 articles, 2 were classified as “fair quality”, 9 as “good quality”, and 1 as “excellent quality” (Table 2). The inter-rater agreement between the two assessors was found to be in excellent agreement, based on Cohen’s kappa coefficient (κ = 0.770; 95% CI: 0.654–0.891). However, only three studies (27.3%) provided justification for their sample sizes. Additionally, the risk of bias assessment conducted for the included studies indicated that 9 studies had a moderate risk of bias [2, 16, 23, 25–27, 56, 57, 59], while 3 studies had a serious risk of bias [11, 58, 60].

**TABLE 2:** Quality assessment scoring of 12 included studies.

Author(s), Year	Reporting					External Validity			Internal Validity					Power

									Bias		Confounding				Total	%	Quality

	Q1	Q2	Q3	Q4	Q5	Q6	Q7	Q8	Q9	Q10	Q11	Q12	Q13	Q14
**Verheul et al., 2024 [26]**	1	1	1	1	1	1	0	1	1	1	1	0	1	0	11/14	78.57%	GQ
**Turner et al., 2024 [27]**	1	1	1	1	1	1	0	1	1	1	1	0	1	0	11/14	78.57%	GQ
**Peng et al., 2024 [57]**	1	1	1	1	1	1	0	1	1	1	1	0	0	0	10/14	71.43%	GQ
**Lima et al., 2024 [2]**	1	1	1	1	1	1	1	1	1	1	1	0	1	1	13/14	92.86%	EQ
**Uhlrich et al., 2023 [25]**	1	1	1	1	1	0	0	1	1	1	1	0	0	0	9/14	64.29%	FQ
**Kakavand et al., 2025 [58]**	1	0	1	1	1	1	0	1	1	1	1	0	0	0	10/14	71.43%	GQ
**Martiš et al., 2024 [11]**	1	1	1	1	1	1	0	1	1	1	0	1	0	1	11/14	78.57%	GQ
**Schwartz et al., 2024 [60]**	1	0	1	1	1	1	0	1	1	1	1	0	0	0	8/14	57.14%	FQ
**Horsak et al., 2023 [16]**	1	1	1	1	1	1	0	1	1	1	1	0	1	1	12/14	85.71%	GQ
**Horsak et al., 2024a [23]**	1	1	1	1	1	1	1	1	1	1	1	0	1	0	12/14	85.71%	GQ
**Horsak et al., 2024b [56]**	1	1	1	1	1	1	1	1	1	1	1	0	1	0	12/14	85.71%	GQ
**Svetek et al., 2025 [59]**	1	1	1	1	1	1	1	1	1	1	1	0	1	0	12/14	85.71%	GQ

*Note:* FQ: Fair quality; GQ: Good quality; EQ: Excellent quality.

### Publication bias

**FIG. 3:**
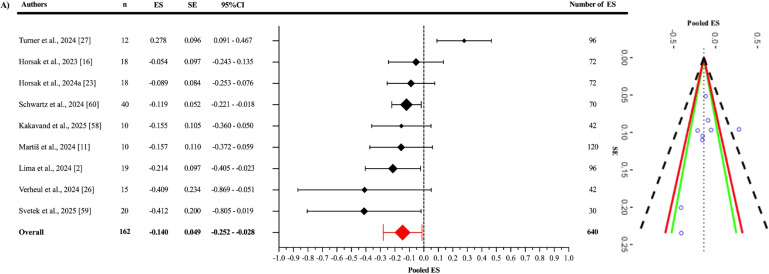
Forest plot of pooled effect size.

**FIG. 4:**
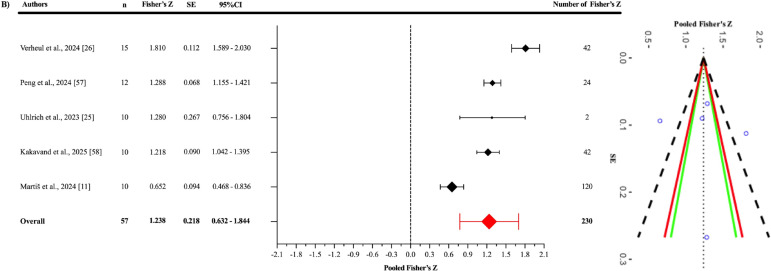
Forest plot of pooled Fisher’s.

**FIG. 5:**
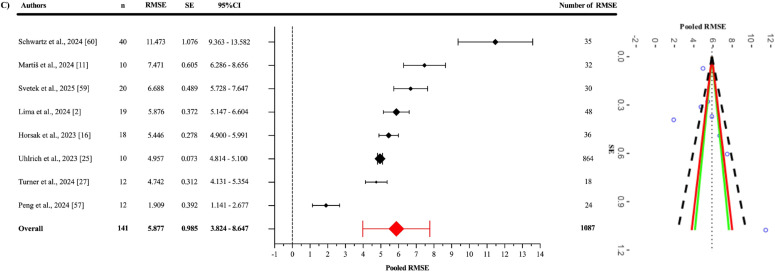
Forest plot of pooled RMSE.

**FIG. 6:**
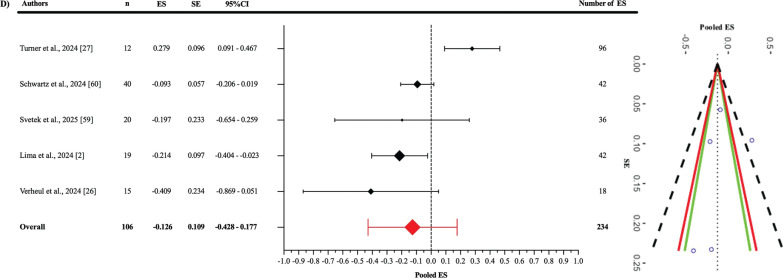
Forest plot of pooled effect size in jump-based subgroup.

**FIG. 7:**
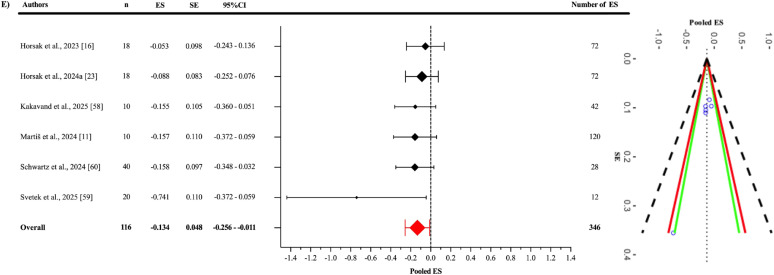
Forest plot of pooled effect size in motion-based subgroup.

Egger’s regression test indicated no evidence of publication bias for the pooled ES (Figure 3), as well as for the jump-based (Figure 6) and motion-based task (Figure 7) subgroups and Fisher’s Z estimates (Figure 4) (all p > 0.05). However, for pooled RMSE (Figure 5), Egger’s regression test revealed statistically significant publication bias (p = 0.003) (Table 3).

**TABLE 3:** Heterogeneity and Egger’s regression test results for publication bias.

Heterogeneity									Publication Bias	
	Model	Q	df	p	%I^2^ (level 1)	%I^2^ (level 2)	%I^2^ (level 3)	Total I^2^	Egger	p
**A**	Three-level	2968.74	639	< 0.001	14.89	0.93	84.18	85.11	-0.009	0.363
**B**	Three-level	1691.11	229	< 0.001	13.68	21.28	65.04	86.32	0.229	0.819
**C**	Three-level	25360.51	1086	< 0.001	0.16	54.01	45.83	99.84	2.841	0.003
**D**	Three-level	1398.78	233	< 0.001	13.68	21.28	65.04	86.32	-0.967	0.333
**E**	Three-level	1568.58	345	< 0.001	17.95	5.71	82.05	87.76	-1.842	0.065

**TABLE 4:** Characteristics of the included studies.

Author(s), Year	Sample And Characteristics	Study Design	Criterion Device(s)
**Verheul et al., 2024 [26]**	15 recreational athletes (M = 9; F = 6) age = 22.4 ± 3.6 years height = 1.75 ± 0.07 m body mass = 77.9 ± 12.6 kg sport participation = 8.9 ± 4.2 h/week experience in sport = 10.1 ± 4.4 years	The study involved three types of jumps: CMJ on a force platform, SJ with a 3-second hold before jumping, and DJ from a 41 cm box. DJs were performed bilaterally and unilaterally on both dominant and non-dominant limbs. All jumps were tested under two conditions: with arm swings or hands fixed on the hips.	A ground-embedded force platform (Kistler 9287CA, 0.6 × 0.9 m, Kistler, Switzerland) sampling at 1000 Hz using Vicon Nexus software (version 2.15, Oxford, United Kingdom).

**Turner et al., 2024 [27]**	12 participants (M = 6; F = 6) age = 18.2 ± 3.3 years height = 179.6 ± 13.2 cm body mass = 75.9 ± 17.6 kg	All participants performed eight trials of a double-leg jump-landing rebound task, single-leg forward hop, and single-leg lateral-vertical hop.	Three-dimensional motion capture 10-camera system (v2.14, Vicon, Nexus, Oxford, UK) sampling at 250 Hz.

**Peng et al., 2024 [57]**	12 participants age = 21.7 ± 1.2 years height = 168.5 ± 7.3 cm body mass = 57.8 ± 7.9 kg	During the gait experiment, they were instructed to walk and run on a designated pathway at a self-selected comfortable speed. The walking and running speeds were quantified as 0.91 ± 0.12 m/s and 1.59 ± 0.2 m/s, respectively.	Three-dimensional motion capture system that consisted of 11 cameras (Vicon, Oxford Metrics Ltd., Oxford, England) and two force plates (OR6, AMTI, Watertown, United States) at sampling frequencies of 200 Hz and 1000 Hz, respectively.

**Lima et al., 2024 [2]**	19 participants (M = 10; F = 9) age = 27.7 ± 4.1 years height = 173.6 ± 9.9 cm body mass = 68.5 ± 11.9 kg	Participants attended two 50-minute sessions at a biomechanics lab, about 7 days apart. Validity was assessed by collecting data with OpenCap and a marker-based system (Vicon) simultaneously in the first session, and test-retest reliability was evaluated across separate days. Each participant performed five tasks in a pre-defined order: single-leg squat, sidestep cut, side hop, single-leg triple vertical hop and double-leg countermovement jump.	Three-dimensional motion capture 11-camera system (Vicon, Oxford, UK) sampling at 200 Hz. Ground reaction forces were collected using three ground-embedded force plates (Advanced Mechanical Technology, MA, USA) sampling at 1000 Hz.

**Uhlrich et al., 2023 [25]**	10 healthy adults (M = 4; F = 6) age = 27.7 ± 3.8 years height = 1.74 ± 0.12 m body mass = 69.2 ± 11.6 kg	OpenCap using two iPhones against marker-based motion capture and force plate analysis in a cohort of ten healthy individuals for several activities (walking with/without trunk sway, squats with/without asymmetric force, sit-to-stands with/without increased trunk flexion, and drop jumps with/without asymmetric landing force).	8-camera motion capture system (Motion Analysis Corp., Santa Rosa, CA, USA) sampling at 100 Hz and 3 force plates (Bertec Corp., Columbus, OH, USA) sampling at 2000 Hz for GRF.

**Kakavand et al., 2025 [58]**	10 healthy adults (M = 5; F = 5) age = 29.5 ± 3.3 years height = 1.76 ± 0.08 m body mass = 70.6 ± 11.8 kg	Study evaluates the performance of marker-based and markerless (OpenCap) motion capture systems in assessing joint kinematics and kinetics during cycling. Pedal reaction forces and crank positions were measured at 250 Hz using Sensix pedals and an encoder. Participants cycled for 20 seconds at two cadences (90 ± 5.0 rpm and 60 ± 5.0 rpm) and three resistance levels (low, normal, high), generating cycling powers between 55 and 352 W at their preferred saddle height.	10-camera motion capture system (Vicon Motion Systems Ltd., Oxford, UK) operating at a sampling rate of 250 Hz.

**Martiš et al., 2024 [11]**	10 participants (M = 6; F = 4) age = 29.7 ± 8.6 years height = 176.6 ± 11.5 cm body mass = 74 ± 13 kg BMI = 23.5 ± 2 kg/m^2^	Walking movements toward and away from the cameras were recorded using marker-based and markerless systems, with 5–7 recordings per participant in each direction. Kinematics were analyzed a musculoskeletal model for the pelvis, hip, knee, and ankle joints. Stance and swing phases, foot progression, lift-off, and landing angles were calculated for each stride. Stride length, walking speed, step length, and step width were measured using heel marker positions and walking direction.	Three-dimensional motion capture 17-camera system (Vicon, Oxford, UK) sampling at 150 Hz.

**Schwartz et al., 2024 [60]**	40 participants (M = 20; F = 20) age = between 18 to 25 sport participation = at least two hours per week	Activities included running 15 m, performing a 45° cutting maneuver, a CMJ, a 30 cm DJ, and a single hop test.	Optoelectronic system (Qualisys, Arqus 9) at 200 Hz.

**Horsak et al., 2023 [16]**	18 healthy participants (three participant were excluded) (M = 9; F = 12) age = 30.2 ± 8.5 years height = 173.0 ± 9.5 cm body mass = 69.6 ± 13.1 kg BMI = 23.2 ± 3.4 kg/m^2^	Participants walked barefoot with minimal clothing at a self-selected speed on a 10 m walkway, performing four gait patterns (physiological, crouch, circumduction, and equinus) in random order. For each participant and walking condition, five left and five right force plate hits were recorded with both, the marker-based and the markerless systems simultaneously.	Three-dimensional motion capture 16-camera system (Nexus, 2.14, Vicon, Oxford, UK) sampling at 120 Hz and three synchronized force plates (AMTI, Watertown, MA, USA) recorded GRF at 1200 Hz.

**Horsak et al., 2024a [23]**	18 healthy participants (three participant were excluded) (M = 9; F = 12) age = 30.2 ± 8.5 years height = 173.0 ± 9.5 cm body mass = 69.6 ± 13.1 kg BMI = 23.2 ± 3.4 kg/m^2^	Participants were instructed by one experienced physiotherapist to walk in a random order with four different gait patterns (physiological, crouch, circumduction, and equinus gait) while simultaneously undergoing marker-based and markerless 3D gait analysis.	16-camera motion capture system (Nexus, 2.14, Vicon, Oxford, UK) was used to record the trajectories of 57 skinmounted markers at 120 Hz.

**Horsak et al., 2024b [56]**	19 healthy participants (M = 12; F = 7) age = 35 ± 11 years BMI = 24.1 ± 3.6 kg/m^2^	Participants completed two sessions, a test and a retest, 26 days apart (SD 3). In the first session, they performed a sit-to-stand task and walked at a comfortable speed, once in street wear and once in minimal clothing, both barefoot. Clothing order was counterbalanced. In the retest session, tasks were performed in minimal clothing only.	Not reported.

**Svetek et al., 2025 [59]**	20 ice hockey players. (F = 18; M = 2) age = 21.35 ± 1.5 years height = 1.71 ± 0.08 cm body mass = 71.08 ± 7.42 kg	Participants were verbally instructed how to complete the gait (walking and running), double leg squat, countermovement jump, and drop landing tasks (12-inch wooden box).	10-camera motion capture (Vicon, Oxford Metrics, London, England) sampling at 240 Hz.

**Author(s), Year**	**OpenCap**	**Criterion Device(s)**	**OpenCap**	**r / R^2^**	**MAE**	**RMSE**		**ICC**	**SEM**	**MDC**
			
		**Mean ± SD**	**Mean ± SD**			**Mean**	**SD**
**Verheul et al., 2024 [26]**	Three iPads (iPad Pro 11-inch, 4^th^ generation, OS version 16.2, Apple, USA) sampling at 240 Hz	✓	✓	✓	⦸	⦸	⦸	⦸	⦸	⦸
	OpenCap was sampled at 60 Hz using two commercial smartphones (iPhone 12 SE, Apple Inc., Cupertino, CA, USA).	✓	✓	✓	✓	✓	✓	⦸	⦸	⦸

**Peng et al., 2024**[**57]**	Two iPhone devices were used at a frame rate of 60 Hz.	⦸	⦸	✓	⦸	✓	⦸	⦸	⦸	⦸

**Lima et al., 2024 [2]**	Two mobile devices (iPhones XS and 11, Apple, USA) were used.	✓	✓	⦸	⦸	✓	✓	✓	⦸	✓

**Uhlrich et al., 2023 [25]**	Five smartphones (iPhone 12 Pro, Apple Inc., Cupertino, CA, USA).	⦸	⦸	✓	✓	✓	⦸	⦸	⦸	⦸

**Kakavand et al., 2025 [58]**	Four smartphones (iPhone 12 Pro, Apple Inc., Cupertino, CA, USA) at a sampling rate of 60 Hz.	✓	✓	✓	⦸	⦸	⦸	⦸	⦸	⦸

**Martiš et al., 2024 [11]**	Two iPhone cameras: an iPhone 12 and an iPhone 14 at a sampling rate of 60 Hz.	✓	✓	✓	⦸	✓	⦸	⦸	⦸	⦸

**Schwartz et al., 2024 [60]**	Two iPads at a sampling rate of 60 Hz.	✓	✓	⦸	⦸	✓	✓	⦸	⦸	⦸

**Horsak et al., 2023 [16]**	Two iOS smartphones (iPhone 11 and 12 Pro) at a sampling rate of 60 Hz.	✓	✓	⦸	⦸	✓	✓	⦸	⦸	⦸

**Horsak et al., 2024a [23]**	Two iOS smartphones (iPhone 11 and 12 Pro) at a sampling rate of 60 Hz.	✓	✓	⦸	⦸	⦸	⦸	⦸	⦸	⦸

**Horsak et al., 2024b [56]**	Two iOS smartphones (iPhone 12 mini and 13 Pro) at a sampling rate of 60 Hz.	⦸	⦸	⦸	⦸	⦸	⦸	✓	✓	✓

**Svetek et al., 2025 [59]**	The OpenCap system (two iOS devices (iPad Air, Apple, Inc) sampling at 60 Hz.	✓	✓	⦸	⦸	✓	⦸	⦸	⦸	⦸

*Note:* F: Female; M: Male; SD: Standard deviation; r: Correlation coefficient; R^2^: Coefficient of determination; MAE: Mean absolute error; RMSE; Root mean square error; ICC: Intraclass correlation coefficient; SEM: Standard error of measurement; MDC: Minimal detectable change; CMJ: Countermovement jump; SJ: Squat jump; DJ: Drop jump.

### Synthesis of results

#### Criterion validity

As a result of the three-level meta-analysis, the pooled ES between OpenCap and criterion devices (Figure 3) was found to be statistically non-significant and slightly negative (ES = -0.140; 95% CI = -0.252 to -0.028; p = 0.021). The Cochran’s Q statistic revealed a statistically significant level of heterogeneity among the studies (Q_639_ = 2968.74; p < 0.001). This heterogeneity was interpreted as “high”, with an I^2^ value of 85.11%. The variance levels contributing to the heterogeneity for OpenCap were as follows: 14.89% for level 1, 0.93% for level 2, and 84.18% for level 3 (A; Table 3). The sensitivity analysis showed that after excluding the study of Martis et al. [11], the ES remained unchanged (ES = -0.140); however, it lost its statistical significance (p = 0.063) [I^2^ = 85.06% (14.95%; 2.59%; 82.47%)]. The sensitivity analysis showed that the results were stable. Additionally, the risk of publication bias was assessed by examining the symmetry of the funnel plot, and the findings were confirmed by the extended Egger’s test, as presented in Table 3 (p = 0.363).

When examining the Fisher’s Z values (Figure 4), the pooled effect was found to be significant and showed a good to excellent positive correlation (r = 0.845; 95% CI = 0.559–0.951; p = 0.005). Statistically significant heterogeneity was detected among the studies (Q_229_ = 1691.11; p < 0.001). This heterogeneity was interpreted as “high”, with an I^2^ value of 86.32%. The variance levels contributing to the heterogeneity for OpenCap were as follows: 13.68% for level 1, 21.28% for level 2, and 65.04% for level 3 (B; Table 3). Additionally, the risk of publication bias was assessed by examining the symmetry of the funnel plot, and the findings were confirmed by the extended Egger’s test, as presented in Table 3 (p = 0.819).

The pooled RMSE value (Figure 5) between OpenCap and the criterion devices was found to be 5.877° (95% CI = 3.985–7.770°; p = 0.001). Statistically significant heterogeneity was detected among the studies (Q_1086_ = 25360.51; p < 0.001). This heterogeneity was interpreted as “high”, with an I^2^ value of 99.84%. The variance levels contributing to the heterogeneity for OpenCap were as follows: 0.16% for level 1, 54.01% for level 2, and 45.83% for level 3 (C; Table 3). The sensitivity analysis, conducted by excluding the study of Schwartz et al. [60], showed that the RMSE value decreased to 5.197° (95% CI = 3.707°–6.688°; p = 0.001) and retained its statistical significance [I^2^ = 96.75% (0.25%; 43.34%; 56.41%)]. The sensitivity analysis showed that the results were stable. Additionally, the risk of publication bias was assessed by examining the symmetry of the funnel plot, and the findings were confirmed by the extended Egger’s test, as presented in Table 3. Egger’s test indicated potential asymmetry (p = 0.003), while Duval and Tweedie’s “trim and fill” procedure was applied to identify the impact of missing studies and to perform necessary adjustments. This procedure suggested that two missing studies might be added, and in the scenario where these two missing studies are included, the pooled RMSE would reach 4.940° (95% CI: 2.870–7.010). This value was still statistically significant, and I^2^ was 95.3%.

### Subgroup synthesis of results

#### Criterion validity of jump-based tasks

In the analysis of the jump-based tasks subgroup (Figure 6), the pooled ES between OpenCap and the criterion devices was found to be statistically non-significant and slightly negative compared to the criterion devices (ES: -0.126; 95% CI = -0.428–0.177; p = 0.312). A statistically significant level of heterogeneity was detected among the studies in this subgroup (Q_233_ = 1398.78; p < 0.001). This heterogeneity was interpreted as “high”, with an I^2^ value of 86.32%. The variance levels contributing to the heterogeneity for OpenCap were as follows: 13.68% for level 1, 21.28% for level 2, and 65.04% for level 3 (D; Table 3). Additionally, the risk of publication bias was assessed by examining the symmetry of the funnel plot, and the findings were confirmed by the extended Egger’s test, as presented in Table 3 (p = 0.333).

#### Criterion validity of motion-based tasks

In the analysis of the motion-based tasks subgroup (Figure 7), the pooled ES between OpenCap and the criterion devices was found to be statistically significant but slightly negative and trivial (ES = -0.134; 95% CI = -0.256 to -0.011; p = 0.0379). A statistically significant level of heterogeneity was identified among the studies analysed in this subgroup (Q_345_ = 1568.58; p < 0.001). This heterogeneity was interpreted as “high”, with an I^2^ value of 87.76%. The variance levels contributing to the heterogeneity for OpenCap were calculated as follows: 17.95% for level 1, 5.71% for level 2, and 82.05% for level 3 (E; Table 3). In the sensitivity analysis, when the study by Martis et al. was excluded [11], the ES changed only slightly, to -0.133 (95% CI = -0.273 to -0.007) and lost its statistical significance (p = 0.058) [I^2^ = 87.55% (20.11%; 7.68%; 79.87%)]. Additionally, the risk of publication bias was assessed by examining the symmetry of the funnel plot, and the findings were confirmed by the extended Egger’s test, as presented in Table 3 (p = 0.065).

## DISCUSSION

This systematic review and three-level meta-analysis aimed to evaluate the criterion validity of the OpenCap markerless motion capture system and to systematically interpret its reliability based on the limited evidence available in the literature. A total of 12 studies were deemed eligible for the systematic review, with 11 (n = 184) providing sufficient data to be included in the meta-analytic synthesis of criterion validity. The overall methodological quality of the individual studies included in the meta-analysis was rated as “fair”, “good”, or “excellent”.

The main findings of this review are as follows: (1) OpenCap has potential to provide valid and acceptable kinematic data across different movement tasks, but heterogeneity between studies limits generalizability. (2) The pooled RMSE was 5.877°, decreasing to 5.197° after excluding one study [60], while trim-and-fill suggested an adjusted value of 4.940°, which should be interpreted with caution due to high heterogeneity. (3) The correlation with criterion devices was good to excellent (r = 0.845). (4) The pooled ES was trivial, and significance was lost when a single study [11] was excluded, showing sensitivity to individual datasets. (5) Subgroup analyses indicated trivial effects in both task types, with motion-based tasks significant and jump-based tasks more consistent. (6) Reliability evidence was generally good to excellent, but some tasks (e.g., trunk rotation or knee flexion/extension in dynamic hops and cuts) showed low reliability, indicating reduced consistency in complex or high-velocity movements.

### Criterion validity

The criterion validity of the OpenCap markerless motion capture system was evaluated through two complementary approaches: (1) agreement/accuracy with criterion devices, determined by RMSE, and (2) correlation with criterion devices, assessed using Fisher’s Z-transformed correlation coefficients. In addition to these, Hedges’ g ESs were calculated.

All studies included to evaluate OpenCap’s criterion validity reported RMSE values. The pooled RMSE for OpenCap, when compared to criterion devices, was calculated as 5.877°, indicating that the system generally has an acceptable margin of error in kinematic measurements. However, this value decreased to 5.197° when the study by Schwartz et al. was excluded [60] in the sensitivity analysis, suggesting that the results may be sensitive to specific studies. Heterogeneity was interpreted as “high”, with an I^2^ value of 99.84%, and this variance was observed to largely stem from Level 2 (54.01%) and Level 3 (45.83%) contributions. This indicates that agreement/accuracy outcomes, as a component of criterion validity, are influenced by inter-study differences (e.g., movement types, measurement conditions, and methodological approaches) and intra-study variations (e.g., different angles).

From a clinical perspective, the integration of markerless motion capture systems into clinical applications depends on their ability to measure human biomechanics accurately and precisely. Researchers evaluating motion capture systems have suggested that an error of 5° or less is considered acceptable [61, 62]. The pooled RMSE value obtained from OpenCap is comparable to the acceptable error levels reported in other markerless motion capture systems [14, 18, 49, 63]. Song et al. compared a commercially available system (Theia3D) with a marker-based motion capture system and reported an RMSD value of 6.8° for the countermovement jump (CMJ), and 9.1° across hip, knee, and ankle angles overall [14]. Similarly, Kanko et al. evaluated the validity of Theia3D by comparing it with a marker-based motion capture system during walking, finding a mean RMSD value of 6.1° across hip, knee, and ankle angles [18]. Turner et al. conducted a study across three different movements (a jump-landing-rebound, single-leg hop, and lateral-vertical hop), revealing that the mean RMSE value for OpenCap across all trials ranged between 2.39° and 6.87°, with an overall mean RMSE of 4.4° [27].

Lima et al., in a concurrent validation study involving five different movements (CMJ, single-leg triple vertical hop, single-leg squat, lateral step-cut, and lateral hop tasks), calculated a mean RMSE value of 6.3° ± 3.5 across all tasks and joint angles, with values ranging from 1.9° (95% CI: 1.4°–2.4°) to 15.7° (95% CI: 13.5°–17.8°). In this study, tasks requiring greater hip flexion, such as the CMJ jump (8.6°), landing phase (9.5°), and squat (12.2°), exhibited lower validity compared to tasks with less hip flexion, such as the lateral step-cut (6.1°) and lateral hop (5.7°) [2]. Furthermore, the results of this study align with findings reported by Uhlrich et al., who identified an RMSE value of approximately 5° for the same joint angles during walking and squat [25]. In a recent validation study on gait patterns, including physiological, crouch, circumduction, and equinus, Horsak et al. found unexpectedly high RMSE values for knee flexion-extension angles (5.7° for physiological gait; 8.5° for crouch gait). The study indicated an overall mean RMSE of 6.6° across walking tasks [16]. Similarly, Peng et al. showed that OpenCap’s RMSE values during walking and running tasks ranged from 3.05° to 7.08° [57]. The results from this review are consistent with those reported in the literature for other markerless motion capture systems (e.g., Theia3D, Azure Kinect) and with previous OpenCap studies [2, 16, 25, 27]. This indicates that OpenCap generally provides a reliable range of error in kinematic measurements and demonstrates a comparable level of measurement validity among markerless technologies. Also, the occurrence of unexpectedly high errors in certain tasks (such as knee flexion-extension angles) suggests that OpenCap’s performance may vary depending on the type of movement and the specific joint angle being analysed. From a clinical perspective, especially if the system is intended to support medical decision-making (e.g., rehabilitation or injury risk assessment), it is important to consider that these error values may exceed the commonly accepted threshold of five degrees. Caution should therefore be exercised when interpreting results, as even small errors in such contexts can lead to critical decisions that may significantly affect individual outcomes.

On the other hand, evidence of publication bias was identified in the criterion validity analysis based on RMSE. Conceptually, this finding may point to a potential file-drawer problem; that is, studies reporting weaker agreement/accuracy may be underrepresented in the literature, leading the published record to present a more optimistic view of OpenCap’s validity than is actually the case. Duval and Tweedie’s trim-and-fill method estimated the addition of two missing studies and reduced the pooled RMSE value to a lower estimate (4.940°). However, it is well known that such imputation-based adjustments are unstable under conditions of heterogeneity (I^2^ = 99.84%), substantial level-2/level-3 variance, and dependence among effect sizes; therefore, the corrected estimate may have limited capacity to compensate for the true extent of publication bias. The high I^2^ value indicates that the variance arises almost entirely from true differences between studies (e.g., task types, methods used, participant populations). In this context, when diverse movement tasks, measurement conditions, and heterogeneous samples are combined, the pooled RMSE essentially reflects an artificial average of highly divergent values and may therefore be misleading when interpreted in isolation. Therefore, the current pooled RMSE value is derived from a very wide range of estimates and may differ from the true level of error. However, future studies conducted with more homogeneous methodological designs (focusing on the same tasks, methods, and comparable populations) could reduce heterogeneity and allow RMSE values obtained in subsequent meta-analyses to be lower and closer to the true estimate than the present one.

In the overall correlation analysis, a statistically significant and good to excellent positive relationship was identified between OpenCap and the criterion devices (r = 0.845). This finding indicates that OpenCap provides results that are consistent and in agreement with those of criterion devices in kinematic measurements, thereby supporting its overall criterion validity. A high level of heterogeneity was observed in the analysis (I^2^ = 86.32%). A substantial portion of this heterogeneity (65.04%) was attributed to methodological differences across studies (Level 3).

Verheul et al. evaluated the validity of OpenCap across various jump tasks, including CMJ, SJ, BDJ, and UDJ. During the CMJ, the correlation for the landing phase peak force (expressed in body weight, BW) was reported as 0.49, with overall moderate to high correlation values observed. However, in the UDJ task, the correlation for the second landing phase peak force (BW) was 0.47, and for the initial contact phase peak force (BW) it was 0.37. Similarly, in the BDJ task, the correlation for the second landing phase peak force (BW) was 0.45. These findings suggest that OpenCap may have limited validity when estimating peak force values [26]. In contrast, Peng et al. reported high correlations between OpenCap and criterion devices for lower limb joint angles during walking. During running, moderate correlations were observed (e.g., 0.53 for hip internal-external rotation and 0.39 for subtalar inversion-eversion). Additionally, high correlations were reported for lower limb joint forces and ground reaction forces during both walking and running. The researchers concluded that OpenCap provides high correlation coefficients and low error levels, particularly in estimating sagittal plane lower limb joint angles and forces, suggesting that the system may serve as a portable and cost-effective alternative in clinical settings [57]. Similarly, Kakavand et al. compared the performance of OpenCap with a marker-based motion capture system for the assessment of cycling biomechanics. The study was conducted with ten healthy adult participants and measured sagittal plane kinematics and dynamics of the hip, knee, and ankle joints using both systems. The results demonstrated very high correlations (r > 0.98) between OpenCap and the marker-based system for joint angles of the hip, knee, and ankle. These findings indicate that the OpenCap is highly consistent in evaluating cycling biomechanics, offering high validity without the need for complex marker placement procedures. The researchers concluded that OpenCap performs comparably to traditional marker-based systems when assessing sagittal plane movements of the hip, knee, and ankle, highlighting its potential as a practical and effective tool for both clinical and research applications. However, they also noted that low correlations were found for moment calculations, particularly at the knee joint, suggesting limitations in OpenCap’s capacity for accurate joint moment analysis. Therefore, caution is advised when interpreting knee joint moment data from OpenCap, and these potential limitations should be considered in practical use [58].

Uhlrich et al. compared the OpenCap system with marker-based systems in terms of biomechanical measurements obtained during tasks such as walking, squatting, sit-to-stand, and drop jump. The system yielded promising results in predicting knee adduction moment during the early stance phase (R^2^ = 0.80) and in estimating the direction of individual-level load changes. However, the absolute error levels in knee moment estimations highlight the need for cautious interpretation, particularly in clinical decision-making processes. The researchers emphasized OpenCap’s potential for capturing biomechanical parameters at low cost and within a short time frame, while also recommending careful use of the system when analysing complex dynamic outputs such as joint moments [25]. Martiš et al. evaluated the limitations of OpenCap in capturing certain angular directions during gait analysis. They compared OpenCap with an optoelectronic system across joint angles including pelvic tilt, hip flexion, knee flexion, and ankle dorsiflexion under different walking strategies. The results revealed that OpenCap demonstrated high correlations in the sagittal plane, particularly for movements such as hip and knee flexion (e.g., r = 0.98 for hip flexion). However, it was noted that in the frontal plane (e.g., pelvis list), correlation values were lower and deviations were more pronounced [11].

In light of the evidence presented, OpenCap demonstrates a high level of agreement with criterion devices, particularly in evaluating joint kinematics in the sagittal plane. The consistently good to excellent correlations observed across studies indicate that OpenCap provides valid and acceptable measurements of hip, knee, and ankle joint angles during tasks such as walking, running, cycling, and jumping. However, lower correlations observed in certain tasks [particularly in frontal plane movements (e.g., pelvis) and more complex dynamic variables (e.g., peak force and knee moment)] suggest that the system may have limited agreement in these areas. Therefore, when conducting assessments using OpenCap, it is essential to carefully interpret the results based on the type of task, the plane of movement, and the specific measurement target. Current findings support the system’s potential as a rapid, cost-effective, and portable solution for clinical and field-based applications; however, its performance may vary depending on the biomechanical parameter being assessed and the context in which it is applied.

Similar to the correlation analysis, pooled ES calculations were used to assess measurement discrepancies between OpenCap and criterion devices. According to the included studies, the pooled ES was statistically significant but practically trivial (ES = -0.140; p = 0.021). However, when the study by Martiš et al. was excluded in the sensitivity analysis [11], this significance was lost (p = 0.063), suggesting that the results may be sensitive to specific sample groups. Even in this case, high heterogeneity persisted (I^2^ = 85.11%), with the majority of this variation (84.18%) again arising from methodological differences across studies (Level 3). Although the measurement outcomes obtained from OpenCap have been compared with those from criterion devices, most of these studies have focused on various jump-based movements (e.g., CMJ, SJ) and movement-based tasks such as cycling, gait, walking toward and away from the camera, running, and walking. In addition, these kinematic measures were assessed across different anatomical regions (e.g., pelvic tilt, pelvic list, pelvic rotation, hip flexion, hip adduction, hip rotation, knee flexion, ankle flexion, subtalar angle, lumbar extension). Some studies also included both left and right limbs and analysed movements in the sagittal and frontal planes. This diversity primarily reflects the need to evaluate OpenCap’s validity across a wide range of movements and multi-joint kinematics. Subgroup analyses provide a more detailed understanding of OpenCap’s performance across different movement tasks. In the jump-based tasks subgroup, the pooled ES between OpenCap and criterion devices was practically trivial and not statistically significant. In the motion-based tasks subgroup, the ES was statistically significant but practically trivial. When the study by Martiš et al. was excluded [11], this significance was lost (p = 0.058), once again indicating that the results may be sensitive to the sample included. The fact that both the overall and subgroup effect sizes were practically trivial suggests that OpenCap does not introduce meaningful measurement bias and can be considered a valid system for capturing kinematic data.

### Reliability

One of the principal advantages of markerless motion capture systems is their capacity to reliably collect data in environments with high ecological validity, while simultaneously reducing the requirement for trained specialists. These features provide significant practical benefits, particularly in the context of time-constrained and large-scale field studies, by eliminating the processes of marker placement and removal [56].

Lima et al. investigated the reliability of the OpenCap system for various joint angles during initial contact and peak angles in triple hop, squat, side hop, cut, and countermovement jump tasks. They reported that OpenCap achieved good to excellent test-retest reliability (ICC = 0.77–0.95) for 69% of variables across joints and tasks, which is 18% lower compared to marker-based systems. Comparing average ICC values, marker-based systems recorded 0.82 for peak angles and 0.75 for initial contact angles, while OpenCap recorded 0.72 for peak angles and 0.64 for initial contact angles. When evaluated by specific tasks, seven out of eight peak angles in the side hop and squat tests showed moderate to excellent reliability, but trunk rotation had the lowest reliability in both tasks (ICC = 0.16; ICC = 0.60). On the other hand, low reliability was reported for knee peak flexion/extension angles in the side-step cut (ICC = 0.25) and triple hop (ICC = 0.37) tasks. The side hop test was the only task showing moderate to excellent reliability for all joint angles at initial contact. Accordingly, the findings indicate that OpenCap offers a level of reliability comparable to marker-based systems [2]. In line with this, Wilken et al. also reported reliability for marker-based systems during level-ground walking in healthy individuals, with ICC values ranging from 0.74 to 0.96 [62]. Additionally, OpenCap was found to achieve higher reliability compared to the Microsoft Kinect-based markerless motion capture system reported by Tamura et al. for hip (ICC = 0.72) and knee (ICC = 0.71) angles [64].

Horsak et al. examined the test-retest reliability of OpenCap during walking and sit-to-stand tasks under minimal and street clothing conditions, finding moderate to excellent inter-session agreement, with ICC values ranging from 0.70 for the femur to 0.97 for the tibia in segment length analysis for the static calibration model. Additionally, they reported that OpenCap’s repeatability under similar clothing conditions was acceptable to good [56]. Keller et al. also found that clothing had no clinically significant effect on kinematic outputs in measurements using the Theia system [65]. Furthermore, Horsak et al. noted that kinematic data collected with OpenCap during walking and sit-to-stand tasks were minimally affected by clothing changes, with differences for most variables remaining below 1° [56].

The minimal detectable change (MDC) is an important metric as it represents the smallest change in a measurement that is unlikely to result from random variability [66]. Horsak et al. reported that OpenCap’s MDC values during walking were, on average, 2.5° higher [56] compared to those reported by Wilken et al. for marker-based gait analysis, with the largest differences observed in sagittal trunk, pelvis, and hip parameters [62]. The study found MDC values ranging from 2° to 16°, with the highest values in sagittal trunk, pelvis, and hip parameters. Average SEM and MDC values were 2.2° and 6.0° for walking, and 2.4° and 6.5° for the sit-to-stand task, respectively [56]. Similarly, Kanko et al. reported that the Theia system showed an average variability of 2.5° across all joint kinematic variables during treadmill walking, with the markerless approach demonstrating less variability across multiple sessions compared to marker-based systems [18]. Supporting these findings, Lima et al. examined OpenCap’s MDC values across various tasks and found an average of 11° (range: 3°–36.1°) for peak and initial contact angles. For the triple hop task, an MDC of 23.6° (SEM: 8.5°) was reported for knee peak flexion/extension angle, and for the squat task, an MDC of 9.2° (SEM: 3.3°) was reported for hip peak internal rotation. In comparison, the marker-based system had an average MDC of 9.5° (range: 3.3°–19.3°), approximately 1.5° lower than OpenCap, though OpenCap exhibited a wider MDC distribution across joints and tasks [2].

Current findings show that OpenCap provides reliability values largely comparable to marker-based systems. However, high variability observed in certain tasks and joint angles (e.g., trunk rotation, and knee peak flexion/extension in side-step cut and triple hop tasks) limits the system’s sensitivity to detect small kinematic changes over time, making it challenging to capture subtle movement differences, particularly in clinical populations or during early rehabilitation stages. Although OpenCap produces results similar to marker-based systems for some tasks and joints, its wider MDC distribution in dynamic tasks (especially triple hop and squat) reinforces this limitation. Therefore, the system appears more suitable and practical as an alternative for field-based research rather than small-scale clinical applications.

### Limitations

Despite the comprehensive approach taken in this systematic review and three-level meta-analysis, several limitations must be acknowledged. First, the relatively small number of included studies (n =12) may limit the generalizability of the findings. While the sample size is sufficient for a meta-analytic synthesis, the restricted pool of studies and participants (predominantly young, healthy individuals aged 18–35 years) may not fully represent the broader population, including older adults, clinical populations, or individuals with movement impairments, who are often the target of motion capture applications in rehabilitation and clinical settings. This demographic homogeneity could influence the applicability of OpenCap’s criterion validity in more diverse contexts. Second, the sensitivity analyses revealed that the exclusion of specific studies (e.g., [11] and [60]) altered the statistical significance or magnitude of the results. This sensitivity to individual studies underscores the influence of outliers or methodological outliers and highlights the need for more standardized protocols in future research to reduce variability and enhance the stability of findings. Finally, none of the included studies investigated OpenCap’s performance in populations with altered biomechanics, such as individuals with musculoskeletal disorders, neurological impairments, or post-surgical rehabilitation conditions. Future research should address different age groups, clinical populations, and performance levels to comprehensively evaluate the validity and reliability of the OpenCap system across diverse real-world and clinical contexts. These limitations collectively suggest that while OpenCap demonstrates promising validity as a markerless motion capture system, the current evidence base is not yet comprehensive or uniform enough to support unequivocal recommendations for its widespread adoption across all clinical and sports applications.

## CONCLUSIONS

This systematic review and three-level meta-analysis included a total of 12 studies (n = 203), 11 of which (n = 184) were incorporated into the meta-analytic synthesis of criterion validity. The results indicate that OpenCap, a smartphone-based markerless motion capture system, can provide valid and acceptable kinematic measurements compared to criterion devices. In terms of reliability, based on the limited number of available studies, test-retest consistency generally ranged from moderate to very good across many joint angles and tasks, although marked variability was observed in certain task-joint combinations. MDC indicators further support these results; the wider MDC distributions observed in dynamic tasks (particularly the triple hop and squat) suggest that OpenCap may have limited sensitivity in detecting small clinical changes.

The pooled RMSE exceeding the frequently cited clinical threshold of 5° (5.877°) indicates potential limitations in sensitivity for certain tasks and joint angles. Publication bias was detected only in the RMSE synthesis, and although the trim-and-fill method predicted two “missing” studies and produced a lower estimate (4.940°), it should be noted that such imputations are unstable under conditions of extreme heterogeneity and dependence among ESs. Therefore, the pooled RMSE is derived from a very wide range of values and may not fully reflect the true level of error. On the other hand, the high I^2^ value indicates that the pooled RMSE, obtained by combining different task types, measurement conditions, and heterogeneous samples, essentially reflects an artificial average of highly divergent values. This suggests that OpenCap’s performance may not be fully represented and could vary substantially depending on the specific task, joint angle, and methodological conditions analysed.

The sensitivity of the findings to individual studies and the predominance of young/healthy samples limit the generalizability of the results to clinical and older/atypical populations. For future research, preregistered protocols, comprehensive and transparent reporting (including null/negative findings), the reporting of prediction intervals, and validation in clinical/older populations should be prioritized. In addition, although the pooled ES remained trivial in magnitude, statistical significance was lost when a single study was removed, indicating that the overall validity effect is fragile.

In conclusion, OpenCap can produce valid and acceptable kinematic measurements under field conditions and offers considerable potential as a cost-effective motion analysis solution. However, the current evidence base suggests that the pooled RMSE value, derived from a very wide range of estimates across different joints and tasks, may not fully reflect the true level of error. Therefore, further research with methodological standardization and clinically more representative samples is essential to ensure that the system can provide consistent, precise, and clinically meaningful outcomes. Future studies should be conducted with samples that include older adults, clinical cohorts (e.g., post–anterior cruciate ligament reconstruction, neurological populations), different BMI levels, and a balanced representation of women and men. Given the limited evidence on reliability, comparative designs assessing inter-/intra-session, inter-rater, and inter-device consistency may be planned; moreover, SEM and MDC values should be reported by joint/task and interpreted in relation to the minimal clinically important difference (MCID) or smallest worthwhile change (SWC). It is also important to evaluate performance under field conditions (clothing, surface, lighting) and to systematically test validity in diverse dynamic movements involving rapid changes of direction, landing, plyometric actions, and deep flexion. These steps will strengthen OpenCap’s capacity to produce clinically meaningful and generalizable outcomes.

## Supplementary Material

Can OpenCap deliver valid and reliable kinematic data for motion analysis? A systematic review and three-level meta-analysis

## Data Availability

All data analyzed in this study are derived from published studies, which are cited in the reference list and available through the publicly accessible journals or databases (PubMed, Scopus, Web of Science, and EBSCO).
